# Smartband-Based Automatic Smoking Detection and Real-time Mindfulness Intervention: Protocol for a Feasibility Trial

**DOI:** 10.2196/32521

**Published:** 2021-11-16

**Authors:** Mark Horvath, Aurora Grutman, Stephanie S O'Malley, Ralitza Gueorguieva, Nashmia Khan, Judson A Brewer, Kathleen A Garrison

**Affiliations:** 1 Department of Psychiatry Yale School of Medicine New Haven, CT United States; 2 Yale University New Haven, CT United States; 3 Department of Behavior and Social Sciences Brown University School of Public Health Providence, RI United States

**Keywords:** smartband, smartphone, smoking, mindfulness, craving, mHealth

## Abstract

**Background:**

Smoking is the leading cause of preventable death in the United States. Smoking cessation interventions delivered by smartphone apps are a promising tool for helping smokers quit. However, currently available smartphone apps for smoking cessation have not exploited their unique potential advantages to aid quitting. Notably, few to no available apps use wearable technologies, most apps require users to self-report their smoking, and few to no apps deliver treatment automatically contingent upon smoking.

**Objective:**

This pilot trial tests the feasibility of using a smartband and smartphone to monitor and detect smoking and deliver brief mindfulness interventions in real time to reduce smoking.

**Methods:**

Daily smokers (N=100, ≥5 cigarettes per day) wear a smartband for 60 days to monitor and detect smoking, notify them about their smoking events in real time, and deliver real-time brief mindfulness exercises triggered by detected smoking events or targeted at predicted smoking events. Smokers set a quit date at 30 days. A three-step intervention to reduce smoking is tested. First, participants wear a smartband to monitor and detect smoking, and notify them of smoking events in real time to bring awareness to smoking and triggers for 21 days. Next, a “mindful smoking” exercise is triggered by detected smoking events to bring a clear recognition of the actual effects of smoking for 7 days. Finally, after their quit date, a “RAIN” (recognize, allow, investigate, nonidentification) exercise is delivered to predicted smoking events (based on the initial 3 weeks of tracking smoking data) to help smokers learn to work mindfully with cravings rather than smoke for 30 days. The primary outcomes are feasibility measures of treatment fidelity, adherence, and acceptability. The secondary outcomes are smoking rates at end of treatment.

**Results:**

Recruitment for this trial started in May 2021 and will continue until November 2021 or until enrollment is completed. Data monitoring and management are ongoing for enrolled participants. The final 60-day end of treatment data is anticipated in January 2022. We expect that all trial results will be available in April 2022.

**Conclusions:**

Findings will provide data and information on the feasibility of using a smartband and smartphone to monitor and detect smoking and deliver real-time brief mindfulness interventions, and whether the intervention warrants additional testing for smoking cessation.

**Trial Registration:**

ClinicalTrials.gov NCT03995225; https://clinicaltrials.gov/ct2/show/NCT03995225

**International Registered Report Identifier (IRRID):**

DERR1-10.2196/32521

## Introduction

Smoking is the leading cause of preventable disease, disability, and death in the United States [1]. Despite large-scale efforts to reduce smoking, the efficacy of clinical interventions for smoking cessation remains low [2]. Although 68% of smokers want to quit, only 7.4% achieve this annually [3], demonstrating an urgent need for more effective treatments [2]. A potentially impactful way to aid smoking cessation is by delivering interventions via mobile health (mHealth) technology such as smartphone apps, which may deliver scalable and effective treatments more efficiently [4-9]. Smartphone apps have numerous advantages for delivering evidence-based treatment for smoking; however, reviews of existing apps report that they have not yet used key potential features to aid smoking cessation [6,7]. In particular, few smoking cessation apps use wearable technologies, most apps require users to self-report smoking, and few apps deliver real-time interventions contingent upon smoking events.

One feature of mHealth technology not yet well-integrated into available smoking cessation apps is data from wearables. Many behavioral treatments for smoking teach smokers to self-monitor their smoking habits, including when, where, and how often they smoke, to learn to identify their smoking patterns and triggers. Wearable technologies such as smartbands are being developed to advance current tracking methods by automatically monitoring and detecting smoking and notifying smokers of smoking events in real time. This feature alone has been found to reduce smoking as compared to a control in a pilot trial [10], a finding consistent with early studies indicating that self-monitoring of smoking behavior alone could reduce smoking [11] particularly among smokers motivated to quit [12]. The ability to accurately track smoking is a critical issue as smokers may underestimate or deny smoking [13], and discordance between detected and self-reported smoking rates can negatively impact clinical trials and population studies that are used to allocate resources and set health priorities [14]. Automatic monitoring and detection of smoking also enables triggering real-time interventions contingent upon detected smoking events. Furthermore, data from wearables can be used to identify individualized profiles of smoking behavior to deliver real-time interventions targeted to *predicted* smoking events. Individual differences in smoking behavior in daily smokers can be characterized by regular patterns of smoking frequency [15], driven by nicotine dependence and other affective and situational factors [16,17]. Therefore, this study tests the feasibility of using a smartband [10] to automatically monitor and detect smoking, deliver real-time interventions triggered by smoking events, and deliver real-time interventions targeted to predicted smoking events according to individualized smoking profiles.

This study uses smartband and smartphone technology to deliver brief mindfulness interventions for smoking cessation. Mindfulness has been defined as the awareness that arises when paying attention in the present moment, on purpose and nonjudgmentally [18]. Mindfulness has been operationalized in research to include maintaining attention on one’s immediate experience and cultivating an attitude of acceptance or nonjudgement toward one’s experience [19]. Mindfulness training typically involves the practice of attention regulation, body awareness, and emotion regulation [20]. For smoking cessation, mindfulness training may help smokers learn to work mindfully with cues, cravings, and affective states that trigger smoking. Smokers may learn to become aware of cues and triggers, pay attention to cravings as they arise, and accept their experience and learn to ride out the cravings rather than to react by smoking [21]. Previous studies indicate that mindfulness training may reduce cigarette craving and withdrawal [22,23], aid in smoking cessation [24,25], and support recovery from lapses following a quit attempt [26]. Furthermore, mindfulness training for smoking cessation delivered by a smartphone app has been demonstrated to be feasible and found to lessen the association between craving and smoking across treatment versus a comparator app [27] and to reduce the neural response to smoking cues and related cigarette consumption [28]. The ability to deliver mindfulness interventions for smoking in context, at the critical moment when smoking or craving occurs, may boost efficacy. Furthermore, brief mindfulness practices may be efficient, accessible, and feasible to learn [29]. Brief mindfulness interventions for smoking have been associated with significant reductions in negative affect, cravings, withdrawal symptoms, and smoking behavior [22,30,31], and with significant increases in mindfulness [29] and have been correlated with outcomes as a component of larger mindfulness training programs for smoking cessation [30,32].

Building upon the extant literature, this study tests the feasibility of using a smartphone and smartband to deliver brief mindfulness exercises to intervene with craving and smoking in real time. This trial tests a three-step intervention among daily smokers making a quit attempt. First, a smartband is used to monitor and detect smoking and notify smokers of smoking events in real time to bring awareness to their smoking behavior and triggers. Next, a “mindful smoking” exercise is triggered by detected smoking events to bring awareness to the present moment effects of smoking—the physical sensations, emotions, and thoughts that result from smoking [21,33]—to begin a process of disenchantment with smoking [21,34]. Last, a “RAIN” (recognize, allow, investigate, nonidentification [21]) mindfulness exercise is targeted to predicted smoking events according to individualized smoking profiles to help smokers work mindfully with cravings rather than to react by smoking [27,32]. The goals of this feasibility trial are to establish that treatment can be delivered per protocol, participants will adhere to treatment, and treatment will be acceptable, prior to a larger clinical efficacy trial. This study will evaluate treatment fidelity, adherence, and acceptability, using outcomes consistent with guidelines and prior studies [35,36].

## Methods

### Overview

A feasibility clinical trial funded by the National Center for Complementary and Integrative Health will be conducted. Ethical approval for the procedures of this trial was obtained from the Yale University Institutional Review Board. All participants will provide online informed consent.

### Setting

The study will be conducted by smartband and smartphone, and be online and optimized for mobile phones. Treatment delivery will be smartphone- and smartband-based.

### Participants

Participants (N=100) will be eligible if they are 18 years or older, smoke at least 5 cigarettes per day (CPD) for at least 2 years, own an iPhone or Android, are fluent in English as intervention content was only available in English at study onset, and are motivated to quit as indicated by a score of at least 4 of 5 on one item of the Action subscale of the Readiness to Change Questionnaire, “I am trying to smoke less than I used to” from 1 (strongly disagree) to 5 (strongly agree) [37].

### Recruitment

Participants will be recruited using online advertising (eg, Facebook ads) and directed to a study website and online screening survey. This recruitment method has been used previously to enroll 505 participants and randomize 56 participants per month in a similar smartband-based clinical trial [27]. This approach should have broad reach. The study website will link to a short screening survey on Yale Qualtrics Survey Tool. The survey will notify an individual if they are eligible or not eligible. If eligible, they will be asked for their contact information and directed to an online informed consent form. Those who provide contact information and online informed consent will receive an email with a copy of the consent form. Participants will then be asked to respond by text to confirm their interest in enrolling in the study and to schedule a short video call with a researcher for onboarding to the study technology. To finalize enrollment, participants must complete the onboarding session.

### Smartband Technology

The smartband used in this study is developed by Somatix, Inc [10] and uses a machine learning algorithm to identify the hand-to-mouth gestures that characterize smoking a cigarette and differentiate these from other similar hand gestures for a given individual. Briefly, raw data are collected from the accelerometer and gyroscope sensors, and following data stabilization and noise filtering, the algorithm determines which movements being performed by the individual signify smoking. Upon smoking detection (3-4 puffs) the user is asked to confirm or deny smoking on the associated smartphone app.

### Onboarding

Upon SMS text message confirmation of interest in enrollment, participants will be shipped the study equipment (smartband, charger, brief instructions, prepaid return envelope). Upon receipt of study equipment, a researcher will conduct an onboarding session with the participant by video call. The onboarding session will walk the participant through using the smartband and smartphone app, including charging and setting up the smartband, pairing and using the smartband and app, and reviewing study instructions. At the end of the onboarding session, participants will be sent a reminder of the study instructions by email. That email will also include troubleshooting tips to use as needed during the trial. Additionally, the email will include a recommendation for quit smoking medications [6]. After onboarding, participants will be SMS text messaged a link to the baseline survey to begin the study. Treatment starters will be defined as completing onboarding and wearing the smartband for 1 day.

### Retention Strategies

Treatment retention will be defined as wearing the smartband on 75% (45/60) of treatment days. Retention rates will be ensured by emphasizing the importance of follow-up at study initiation, onboarding, and at each study survey; paying US $10 for onboarding, US $20 for the survey at 21 days, US $10 each for surveys at 28 and 60 days, and US $1 per day for wearing the smartband for 12 waking hours; paying for surveys immediately upon survey completion; and paying a US $50 completion bonus at the end of the study for completing all parts of the study.

### Intervention

**Figure 1:**
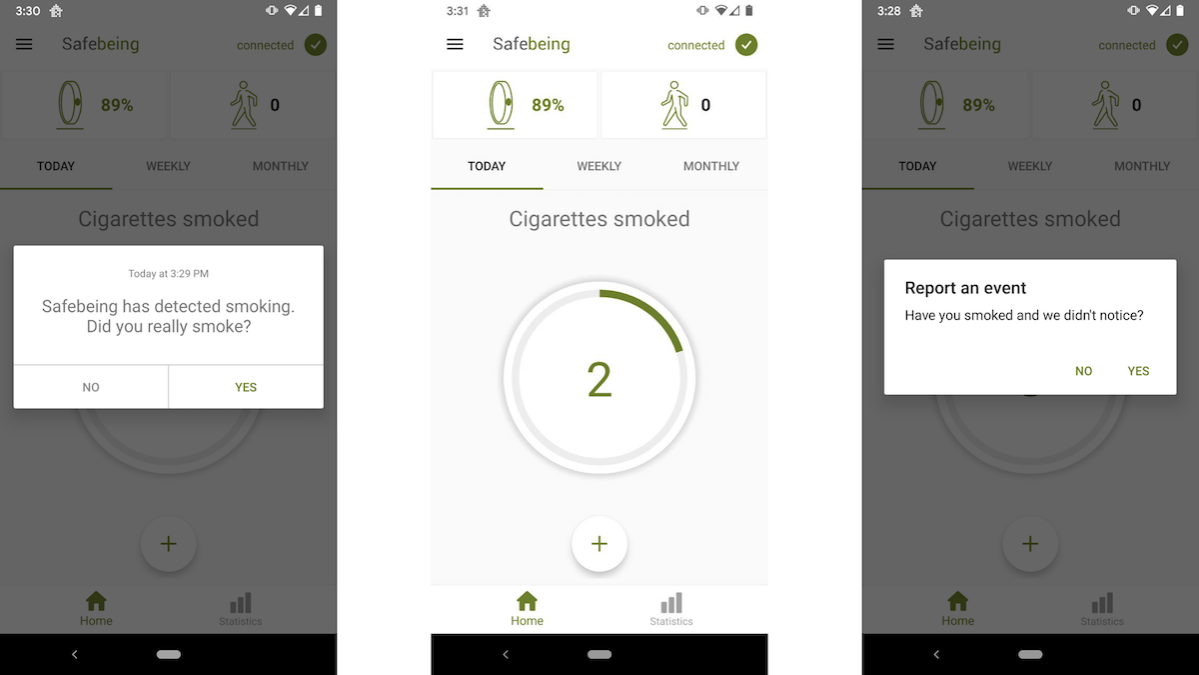
Smartband app smoking notification and tracking. Panel 1: Smoking is detected in real time and a notification is pushed to the user to confirm or deny smoking. Panel 2: Cigarettes smoked per day are displayed on the home page of the app. Panel 2-3: The user can report a smoking event manually if smoking is not detected by the smartband by pressing “+” on the home page and confirming smoking.

**Figure 2:**
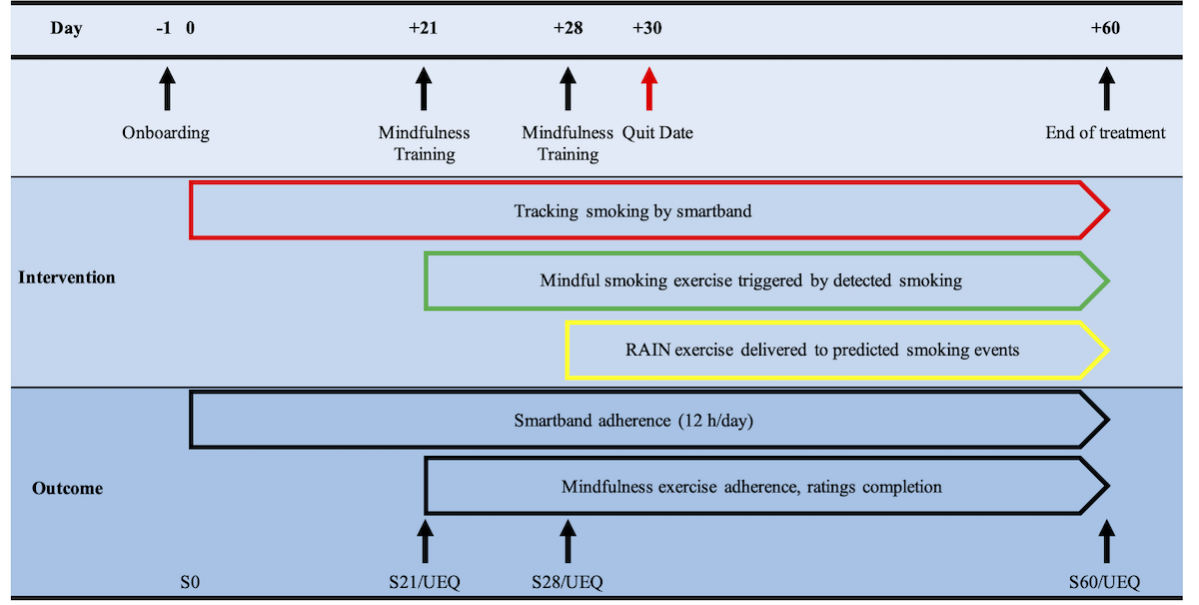
Study timeline. RAIN: recognize, allow, investigate, nonidentification; S: survey; UEQ: User Experiences Questionnaire.

All participants will receive a smartband and smartphone app for the smartband (Figure 1) and take part in a 60-day intervention (Figure 2). For the first 21 days, the smartband will automatically monitor and detect smoking and notify the participant of smoking events in real time to bring awareness to smoking behavior and triggers. When smoking is detected, the smartband will vibrate and the participant will receive a notification on their smartphone app asking them to confirm or deny smoking. The notification will remain until they reply. Alternatively, if a participant smokes and it is not detected by the smartband, participants will be asked to manually report the smoking event on the smartphone app. They will be instructed to smoke as much or as little as they like [31] but will be asked to set a quit date in 30 days. Participants will be instructed to wear the smartband for 12 waking hours per day. They will be automatically sent a reminder notification from the app if they wear the smartband for <12 hours each day. Additional SMS text message reminders will be sent by the study team if they wear the smartband for <12 hours per day for 48 and 72 hours. If they do not wear the smartband at all, they will receive an additional SMS text message, email, or phone call at 48 and 72 hours. Data from this initial 21 days of smoking monitoring and detection will be used to predict individual patterns of smoking for RAIN mindfulness exercise delivery later in the intervention.

At 21 days, participants will be SMS text messaged a link to the 21-day survey. At the end of the survey, they will take part in a brief mindfulness training adapted from Craving to Quit, a smartphone app for mindfulness training for smoking cessation [27,38]. The mindfulness training uses videos and animation to introduce mindfulness, introduces habit formation using an animation, and provides a guided mindful smoking exercise, which helps participants bring awareness to the present moment effects of smoking to gain a clear recognition of the effects of smoking. After the mindfulness training, participants will be instructed that, for the next week, the smartband will continue to automatically monitor and detect smoking, notify them of smoking events in real time, and ask them to confirm or deny smoking. They should continue to manually report any smoking events that are not detected by the smartband. In addition, anytime smoking is detected by the smartband or manually reported, they will automatically receive an SMS text message with a link to the mindful smoking exercise. They can also access mindful smoking anytime by clicking the link in their text messages. They will be instructed to try to use the mindful smoking exercise any time they smoke. After the mindful smoking exercise, they will be asked to rate how helpful they found it. The link will direct the participant to a 2-minute audio-guided mindful smoking exercise with subtitles, followed by an ecological momentary assessment (EMA) rating, “How helpful did you find this exercise?” (visual analog scale [VAS]: “Not at all helpful” to “Very helpful”).

At 28 days, participants will be SMS text messaged a link to the 28-day survey. At the end of the survey, they will take part in another brief mindfulness training adapted from Craving to Quit. The mindfulness training uses videos and animation to introduce the concept of craving, including using an animation with the metaphor of craving as a tantrum toddler (ie, let the toddler cry it out, introduces the concept of urge surfing, and provides a guided mindfulness exercise, RAIN) [21]. This exercise teaches smokers how to work mindfully with triggers to smoke using RAIN. Smokers learn to recognize cues, cravings, or affective states that trigger smoking; allow and accept their experience, however pleasant or unpleasant, without trying to change it; investigate what the experience feels like in the body, emotions, and thoughts; and nonidentification with what is happening from moment-to-moment (RAIN). By openly investigating one’s present moment experiences of craving in a nonjudgmental way, one may learn that cravings are physical sensations, thoughts, and emotions that will subside. After the mindfulness training, participants will be reminded of their quit date, and instructed that, for 30 days after their quit date, the RAIN exercise will be sent to them by SMS text message at the times they typically smoke. They can also access the RAIN exercise anytime by clicking the link in their SMS text messages. They will be instructed to try to use the RAIN exercise any time they have a craving to smoke. RAIN will be delivered according to individualized smoking profiles generated from smoking data from the first 21 days of the intervention. Briefly, a prediction algorithm is run every 5 minutes and if >1 smoking event is detected within 15 minutes of the current time on any day in the 21 days, the answer is true. A text cooldown period is set at 45 minutes such that RAIN will not be sent twice within 45 minutes. These parameters were determined by an initial pilot study (n=8 completers) to provide support but minimize text burden. Before and after the RAIN exercise, they will be asked to rate their craving and affect [39]. After the RAIN exercise, they will also be asked to rate how helpful and timely they found it. The link will direct participants to a 2-minute audio-guided RAIN exercise with subtitles, preceded by rating, “How much are you craving a cigarette right now?” (VAS: “Not at all” to “Very much”) and “How are you feeling right now?” (VAS: “Very bad” to “Very good”), and followed by the same ratings plus “How helpful did you find this exercise?” (VAS: “Not at all helpful” to “Very helpful”) and “How was the timing of this exercise?” (VAS: “Too early” to “Too late”). Additionally, participants will be instructed that the smartband will continue to automatically monitor and detect smoking, notify them of smoking events in real time, and ask them to confirm or deny smoking. They should continue to manually report any smoking events that are not detected by the smartband. Anytime smoking is detected by the smartband or manually reported, they will continue to automatically receive an SMS text message with a link to the mindful smoking exercise.

Throughout the intervention, participants can delay or skip mindfulness exercises by ignoring the SMS text message. They can also access the mindfulness exercises at any time by clicking the link in their SMS text messages. Completion of mindfulness exercises will be time-stamped including onset and offset times for the 2-minute guided audio. To encourage completion of mindfulness exercises, participants will be sent encouraging SMS text messages indicating the number of exercises they completed on the previous day with congratulations or a reminder to use the mindfulness exercises if none were completed. These texts will be sent daily for mindful smoking (week 4) and for the first week of RAIN (week 5), and weekly thereafter for RAIN (weeks 6-8).

At 60 days, participants will be SMS text messaged a link to the 60-day (end of treatment) survey. At the end of the survey, individuals who report 1-week point prevalence abstinence from smoking (up to n=30) will take part in carbon monoxide (CO) monitoring. CO levels will be measured using piCO+ Smokerlyzer breath CO monitors (Bedfont Scientific Ltd). Participants will be shipped the CO monitor, brief instructions, and a prepaid return envelope. They will set up a video call with a researcher (recorded), during which they will use the CO monitor and display the output [27,40,41]. Once a participant has completed the 60-day survey and returned any study equipment, they will be paid their final study payment.

### Measurements

#### Primary Outcome Measures

The primary outcome measures for this feasibility clinical trial are treatment fidelity, adherence, and acceptability across the 60-day intervention.

##### Treatment Fidelity

Treatment fidelity measures will include the following: *whether we can accurately detect smoking events* will be measured as the percent of smoking events detected and the rate of false alarms in the first 21 days of the study during which smokers are wearing the smartband to monitor and detect smoking, are notified of smoking events in real time, and are asked to confirm or deny smoking and to self-report any undetected smoking events. We expect to replicate previous findings of >80% detection and negligible rate of false alarms [10]. *Whether we can deliver mindful smoking triggered by smoking events* will be determined by the percent of mindful smoking exercises correctly triggered by smoking events and the rate of false alarms in the next 7 days (days 21-28) of the study during which any detected or reported smoking event triggers delivery of the mindful smoking exercise by SMS text message. Again, we expect to replicate previous findings of >80% detection and a negligible rate of false alarms [10]. *Whether we can deliver RAIN to predicted smoking events* will be measured as average timeliness ratings in the final 30 days (days 30-60) of the study during which RAIN is delivered to predicted smoking events, and the participant is asked to rate timeliness. Additionally, craving ratings obtained before participants complete RAIN will be used as a proxy for whether we have targeted moments of high craving. We expect on average high timeliness and craving ratings. Ratings will be evaluated as percent of participants and score range (very low, low, moderate, high, very high), with feasibility determined by 75% of participants rating an item as moderate or higher.

##### Adherence

Adherence will be measured as percent of days participants wear the smartband for 12 waking hours during the 60-day study, percent of smoking notifications answered (confirmed or denied) of those sent during the 60-day study, percent of mindful smoking exercises completed of those sent during the mindful smoking period (days 21-28) of the study, and percent of RAIN exercises completed of those sent during the RAIN period (days 30-60) of the study. Based on our pilot data, adherence cut-offs will be 80% of mindfulness exercises completed relative to the participant’s baseline CPD.

##### Acceptability

Acceptability will be measured as mean helpfulness ratings, separately for mindful smoking and RAIN exercises. Again, ratings will be evaluated as percent of participants and score range (very low, low, moderate, high, very high), with feasibility determined by 75% of participants rating an item as moderate or higher. Acceptability will additionally be evaluated from feedback on User Experiences Questionnaires administered as part of the surveys at each time point (21, 28, and 60 days), which ask about the acceptability of the technology (eg, was it easy/difficult to complete onboarding, get technical support, keep the smartband charged, or keep the smartband paired with the smartphone) and intervention (eg, helpfulness of mindfulness exercises; liking of mindfulness exercises; or effect of mindfulness exercises on awareness, craving, and smoking). Average responses for a given rating will be evaluated as percent of participants and score range (very low, low, moderate, high, very high), with feasibility determined by 75% of participants rating an item as moderate or higher. The User Experiences Questionnaire at 60 days also includes items adapted from standardized measures including the User Burden Scale [42], System Usability Scale [43], Mobile Application Rating Scale [44], and the 4-item Acceptability of Intervention Measure [45].

#### Secondary Outcome Measures

The secondary outcome measures will be smoking and abstinence rates. CPD will be measured by self-report at each study survey (baseline, 21, 28, and 60 days) and detected by the smartband. Each study survey will also include the Timeline Followback [46] for the previous study period (between surveys). Additionally, 1-week point prevalence abstinence rates at 60 days will be measured by self-report (60-day survey), detected by smartband (no smoking events for the past 7 days), and based on biochemical verification (<6 parts per million CO; n=30). Continuous abstinence (<5 cigarettes since quit date [47]) will also be measured by self-report.

#### Exploratory Outcomes

Exploratory measures will include survey data collected at baseline, 21, 28, and 60 days (Table 1): demographics, smoking characteristics including the Fagerström Test for Nicotine Dependence [48] and Minnesota Tobacco Withdrawal Scale [49], and the Five Facet Mindfulness Questionnaire short form [50,51] as well as EMA ratings (craving, affect) collected throughout the intervention.

**Table 1:** Study measures.

Domain and measures		Screen	Day 0	Day 21	Day 28	Day 60
**Demographics**						
	Demographics	✓^a^	—^b^	—	—	—
	Socioeconomic status	—	✓	—	—	—
	Language preference	—	✓	—	—	—
	PhenX Gender Identity [52]	✓	—	—	—	—
	PhenX Educational Attainment [52]	—	✓	—	—	—
**Smoking**						
	Smoking Status [47]	✓	✓	✓	✓	✓
	Timeline Followback [53]	—	✓	✓	✓	✓
	Biochemical verification of abstinence	—	—	—	—	✓
**Nicotine dependence**						
	Fagerström Test for Nicotine Dependence [48]	—	✓	—	—	✓
	Minnesota Nicotine Withdrawal Scale [49]	—	✓	✓	✓	✓
**Other treatments**						
	Medication Use Questionnaire	—	✓	✓	✓	✓
	Current e-cigarette use	✓	✓	✓	✓	✓
**Smoking self-efficacy**						
	Self-efficacy Questionnaire [54]	—	✓	✓	✓	✓
	Readiness to change [37]	✓	—	—	—	—
**Mindfulness**						
	Five Facet Mindfulness Questionnaire [51]	—	✓	✓	✓	✓
**User Experiences Questionnaire**						
	User Experiences Questionnaire	—	—	✓	✓	✓
	User Burden Scale [42]	—	—	—	—	✓
	System Usability Scale [43]	—	—	—	—	✓
	Mobile Application Rating Scale [44]	—	—	—	—	✓
	Acceptability of Intervention Measure [45]	—	—	—	—	✓
	Feasibility of Intervention Measure [45]	—	—	—	—	✓
**Other**						
	eHealth literacy [55]	—	✓	—	—	—

^a^Indicates this item is included.

^b^Indicates this item is not included.

### Statistical Analysis

Descriptive statistics of primary and secondary outcome measures will be reported prior to statistical analysis. For treatment fidelity, adherence, and acceptability, we will estimate proportions with 95% CIs. Change in smoking will be assessed using mixed regression models to compare self-reported and smartband-detected CPD, using all available data with focused contrasts on changes from baseline to 60 days. Random effects or structured variance-covariance matrix of the errors will be used to take into account correlations of repeated measures within an individual, and the best-fitting model will be selected based on Bayesian information criterion. We will perform exploratory mediation analysis of the exploratory outcomes to test mechanistic hypotheses related to smoking, craving, affect and mindfulness, and other psychological variables.

## Results

The study was approved by the Yale University Institutional Review Board in March 2019 and funded in May 2019. The development and programming of the study intervention has been completed. All study methods and materials have been developed and manualized. An initial pilot study (n=30) was conducted between November 2020 and January 2021 to test and finalize the study intervention and procedures. Recruitment for the full feasibility trial began in May 2021 and will continue until November 2021 or until enrollment is complete. Data monitoring and management are ongoing for enrolled participants. The final 60-day end of treatment data is anticipated to be collected in January 2022. We expect that all trial results will be available in April 2022.

## Discussion

### Principal Results

This trial is designed to test the feasibility of using a smartband and smartphone to automatically monitor and detect smoking, notify smokers of smoking events in real time, and deliver brief mindfulness interventions to help daily smokers in a smoking quit attempt. We anticipate collecting important data and information regarding innovative features of the intervention including using wearable technology (smartband) to monitor and detect (track) smoking, automatically detecting smoking rather than relying on self-report, using data from wearables to identify patterns of individual smoking behavior over time, delivering interventions contingent on smoking events and targeted to predicted smoking events, and combining novel mHealth technology with an evidence-based mindfulness intervention for smoking cessation.

### Limitations

There are several potential limitations of this study. First, it is possible that data from the initial 21 days of tracking smoking by smartband may not be sufficient to obtain predictable individualized smoking profiles. This is an empirical question—the trial will provide useful data and information on our ability to predict smoking based on 21 days of smartband data. Second, smoking patterns may change over time in response to being monitored for smoking and other factors. Averaging smoking patterns for an individual across 21 days allows us to account for these changes but stay in line with clinical guidelines for setting a quit date at 30 days [56]. Third, additional psychological mechanisms other than craving and affect may impact smoking. Once the feasibility of the approach is established, future studies can include additional EMA ratings to evaluate other key psychosocial factors (eg, anxiety, stress, or activity). Fourth, an alternative design would be to combine the smartband technology with another full-scale treatment for smoking such as the Craving to Quit app [27]. However, it is a challenge in clinical trials of smartphone apps to determine efficacious components of multifeatured apps. This study takes a component approach by testing mindful smoking and RAIN exercises, which also builds upon prior work including our own demonstrating that brief mindfulness interventions can reduce craving and smoking. Last, individuals may use the technology inappropriately, such as wearing the smartband on the hand not used for smoking or not wearing the smartband upon waking up. Although we cannot completely control for missed cigarettes due to these or other factors, tracking smoking by smartband vastly improves smoking estimates beyond self-report, and this study will be one of the first to test how smartband-based smoking detection relates to self-reported CPD. These activities and other factors will also be queried on user experience questionnaires at each time point.

### Conclusions

This study will provide data and information on whether smartphone- and smartband-based smoking monitoring and detection, notification, and delivery of brief mindfulness interventions aid daily smokers in a quit attempt. Feasibility outcomes will be used to inform future randomized clinical trials of the tested technology and intervention.

## Appendix

Multimedia Appendix 1 External peer-review report.

## Abbreviations

CO: carbon monoxide
CPD: cigarettes per day
EMA: ecological momentary assessment
mHealth: mobile health
RAIN: recognize, allow, investigate, nonidentification
VAS: visual analog scale
