# Supplementary material for: Smartband-Based Automatic Smoking Detection and Real-time Mindfulness Intervention: Protocol for a Feasibility Trial
Source: JMIR Res Protoc. 2021 Nov 16;10(11):e32521. doi: 10.2196/32521 (PMC8663689; doi:10.2196/32521)
Supplement: Multimedia Appendix 1 [file resprot_v10i11e32521_app1.pdf]

SUMMARY STATEMENT

PROGRAM CONTACT:
Dr. David Clark
301-827-1916
dave.clark@nih.gov

(Privileged Communication)

Release Date: 12/15/2018
Revised Date:

Application Number: 1 R34 AT010365-01

Principal Investigator

GARRISON, KATHLEEN A.

Applicant Organization: YALE UNIVERSITY

Review Group: ZAT1 PJ (03)
National Center for Complementary and Integrative Health Special Emphasis Panel
Exploratory Clinical Trials of Mind and Body Interventions (MB)

Meeting Date: 11/15/2018
Council: JAN 2019
Requested Start: 04/01/2019

RFA/PA: PAR18-417
PCC: CLARKD

Project Title: Smartband/smartphone-based automatic smoking detection and real time mindfulness intervention
SRG Action: Impact Score:29
Next Steps: Visit https://grants.nih.gov/grants/next_steps.htm
Human Subjects: 30-Human subjects involved - Certified, no SRG concerns
Animal Subjects: 10-No live vertebrate animals involved for competing appl.
Gender: 1A-Both genders, scientifically acceptable
Minority: 1A-Minorities and non-minorities, scientifically acceptable
Children: 1A-Both Children and Adults, scientifically acceptable

Project Year	Direct Costs Requested	Estimated Total Cost
1	225,000	376,875
2	225,000	376,875
TOTAL	450,000	753,750

ADMINISTRATIVE BUDGET NOTE: The budget shown is the requested budget and has not been adjusted to reflect any recommendations made by reviewers. If an award is planned, the costs will be calculated by Institute grants management staff based on the recommendations outlined below in the COMMITTEE BUDGET RECOMMENDATIONS section.
BUDGET MODIFICATIONS

1R34AT010365-01 GARRISON, Kathleen

COMMITTEE BUDGET RECOMMENDATIONS

RESUME AND SUMMARY OF DISCUSSION: This R34 application titled “Smartband/smartphone-based automatic smoking detection and real time mindfulness intervention” is submitted in response to PAR18-417 “Feasibility Clinical Trials of Mind and Body Interventions for NCCIH High Priority Research Topics (R34)” by Yale University Department of Psychiatry with Dr. Kathleen Garrison as the principal investigator. The overall goal of this well-written application is to utilize novel wearable smartphone technology to detect, track and deliver a mindfulness intervention to daily smokers. To this end, the project specifically aims to test the fidelity, adherence and acceptance of the smartband/smartphone-based mindfulness intervention delivered in real time to reduce smoking. The potential impact of this work, if successful, may provide scalable prevention options for smoking cessation, a significant public health problem. The study rationale is based on compelling preliminary work conducted by the study team indicating feasibility and successful recruitment using an app-based mindfulness intervention compared to controls for reduced smoking rates. The panel highlighted the real-world use of wearable technology to intervene on craving and smoking cessation in real time as highly innovative. The panel also acknowledged that the aims were well-matched to capture feasibility outcomes and that the project is likely to deliver data that will inform the design of a future clinical trial. The investigative team is eminently suited for the conduct of this work, including extensive experience in smoking cessation, mindfulness, smartphone apps and clinical trials. However, the panel noted the role of the consultant (Brewer) was unclear and the devoted effort was considered insufficient given the complementary expertise needed to complete the project. Overall, the environment is strong with adequate resources and recruitment capabilities. During the discussion, concerns were raised related to the approach. The panel noted the specific mindfulness strategies selected from preliminary work were not well-justified since the two strategies were shown to have lower engagement by participants. It was also noted that the audio version of the app may introduce a barrier for real world use, which may have contributed to the lower engagement observed and this was not adequately discussed in the application. Other minor issues raised during the discussion involved a limited consideration for smokers with health disparities who are more likely to smoke and may have less access to smartphones as well as an inadequate approach for compensation, which may introduce confounds for completers. However, these minor detractions are outweighed by the highly significant and innovative aspects of the project, the importance of the topic under study, and stellar qualities of the study team overall and the environment, yielding a very sound project with the potential for high impact on the field of substance abuse and mindfulness-based approaches.

DESCRIPTION (provided by applicant): Smoking is the leading cause of preventable death in the US. Effective smoking cessation interventions are available but underutilized. Smoking cessation interventions delivered by smartphone apps are a promising tool for helping smokers quit. Delivery of treatments via smartphone apps may maximize the likelihood of use by smokers and the potential impact on smoking behavior. However, currently available smartphone apps for smoking cessation have not exploited their unique potential advantages to aid quitting. Notably, no available apps utilize wearable technologies; all current apps require users to self-report their smoking; and no apps deliver treatment automatically contingent upon smoking. Therefore, this pilot trial will test the feasibility of using a smartband to detect and track smoking and deliver brief smoking cessation interventions by smartphone app in real time. The interventions to be delivered will be brief mindfulness exercises that have been previously shown to reduce craving and smoking. This trial uses SmokeBeat, a novel mobile technology platform that uses multimodal data from wristband sensors to monitor and detect smoking, notify smokers about their smoking in real time and deliver real time interventions triggered by detected smoking episodes. SmokeBeat also applies machine learning to smoking tracking data to identify individual smoking patterns and deliver real time interventions targeted at predicted smoking episodes. This trial tests a three-step intervention to reduce smoking, in which smokers first become aware of

their smoking and triggers by tracking smoking; then gain a clear recognition of the actual effects of smoking by “mindful smoking”; and finally learn to work mindfully with cravings rather than smoke. Briefly, daily smokers (N=200, ≥5 cig/day) will wear a smartband to detect and notify them of smoking for 21 days and obtain individual smoking profiles; detected smoking will then trigger a “mindful smoking” exercise for the next 7 days leading up to their quit date at 30 days; after which another mindfulness exercise (“RAIN”: recognize, accept, investigate and note cravings rather than smoke) will be delivered prior to each predicted smoking episode according to their individual smoking profile for 30 days post-quit. Aim 1 will be to determine treatment fidelity. Fidelity measures will be: (1) percent of smoking episodes correctly detected; (2) percent of “mindful smoking” exercises correctly triggered by smoking; and (3) users’ real time ratings of how timely “RAIN” was delivered to predicted smoking episodes. Aim 2 will be to determine adherence to treatment. Adherence measures will be: (1) percent of time spent wearing the smartband; (2) percent of smoking notifications answered; (3) percent of ecological momentary assessment (EMA) ratings (e.g., timeliness and others) answered; and (4) percent of mindfulness exercises completed. Aim 3 will be to determine the acceptability of treatment. Acceptability measures will be: (1) average helpfulness ratings after each mindfulness exercise; (2) feedback on user experience surveys. Overall: this project tests a highly innovative technology-based mindfulness intervention for smoking cessation.

PUBLIC HEALTH RELEVANCE: Recent developments in mobile technology suggest new and innovative ways to deliver effective smoking cessation treatments. This project will test the feasibility of using a smartband to automatically monitor and detect smoking and deliver real time mindfulness interventions via smartphone app to reduce smoking. This pilot trial will provide important data and information to inform a larger clinical efficacy trial.

CRITIQUE 1

Significance: 3
Investigator(s): 2
Innovation: 2
Approach: 5
Environment: 2

Overall Impact: This application proposes to examine the ability of a smartband to detect and track smoking behavior while also delivering a mindfulness-based smoking cessation intervention. Participants (N=200) will enroll in the study for a total of 2 months (first 21 days will obtain data to create unique smoking profiles; next 7 days will deliver a “mindful smoking” exercise whenever a participant smokes; next 30 days will deliver the mindfulness exercise “RAIN”). The quit date will occur about 30 days into the study. Aim 1 will determine treatment fidelity (e.g., % smoking episodes correctly detected, accuracy in the delivery of mindfulness exercises); Aim 2 will examine treatment adherence (e.g., % time wearing smartband, % EMAs responded to, % mindfulness exercises completed); Aim 3 will examine treatment acceptability (e.g., helpfulness of mindfulness exercises). Overall, this is a very innovative project that will be among the first to deliver a smoking cessation intervention via a wearable device and phone app. Strengths of the study include a strong scientific premise for the intervention and use of technology, feasibility of recruitment and retention, specific aims that capture clear feasibility outcomes, the ability to use these data to inform a future clinical trial, and the investigative team. Primary limitations that somewhat lessen enthusiasm for the application include the selection of the two mindfulness exercises, a lack of clearly outlined benchmarks to evaluate each sub-question within the aims, and compensation for the completion of the mindfulness exercises. If these limitations can be addressed, this application has the potential to move the field forward regarding scalable cessation interventions.

1. Significance:

Strengths

- Current smoking cessation rates are low and utilizing technology to reach more smokers has the potential to aid more quit attempts.
- Mindfulness-based interventions and strategies can break the link between craving and smoking, and negative affect and smoking. Thus, providing mindfulness-based strategies via technology during a quit attempt makes sense.
- Successful completion of this project will inform the future use of both technology and brief mindfulness interventions in the context of smoking cessation.
- Next steps will include an RCT to evaluate the efficacy of the smartband mindfulness intervention when compared to an active comparison control.

Weaknesses

- Although previous research has shown that mindfulness-based interventions are efficacious for smoking cessation, very little work has been conducted to understand how mindfulness strategies delivered via technology (i.e., not within a group-based, in-person treatment context) will work. And although Craving to Quit (developed by the investigators) was a smartphone-delivered mindfulness intervention, it appears to be quite comprehensive in the delivery and training of mindfulness, and despite this, there were no significant differences between groups on abstinence. The main concern here is related to the "dose" of mindfulness participants will receive in the current study via very brief strategies delivered via smartphone.
- The investigators could also consider a micro-randomized trial as a future, larger study to evaluate the efficacy of whether delivering a specific mindfulness strategy at a given moment in time (e.g., mindful smoking when smoking a cigarette) is ideal. This study design would allow the investigators to further tailor and optimize their intervention.

2. Investigator(s):

Strengths

- The investigative team is highly qualified to conduct the current project. The principal investigator, Dr. Garrison, has expertise in addiction, mHealth, and mindfulness. She currently has a Varela grant from Mind and Life on these topics. Although she is early career, her co-investigators are well-established senior investigators with relevant expertise.
- The team has experience successfully recruiting this population in previous trials and has published study findings.

Weaknesses

- Dr. Brewer's effort is listed as 0%, and he will be available on an as-needed basis. Given the amount of knowledge Dr. Brewer brings to the project, combined with Dr. Garrison's early career status, the specific amount of effort allocated to Dr. Brewer over the project period is insufficient.

3. Innovation:

Strengths

- This application is very innovative, as it will utilize mHealth to provide a brief cessation intervention at specific moments during a quit attempt that are likely to aid cessation.
- Identifying unique smoking profiles to intervene upon during a quit attempt is innovative.
- Given the general increase in technology utilization, combined with the need to increase smoking cessation in the population, this project could develop a scalable intervention that could ultimately decrease smoking-related morbidity and mortality.

Weaknesses

- No major weaknesses noted.

4. Approach:

Strengths

- The three Aims are ideal to evaluate the feasibility of the current intervention.

- The ability to recruit the intended population within the timeframe is a strength, based on previous work by the investigators.
- Plans to recruit N=300 to retain 200 participants is appropriate.
- The procedures for data management and analysis fit with the aims of the study.

Weaknesses

- The two mindfulness exercises that will be provided to participants – mindful smoking and RAIN – were underutilized by participants in the prior trial. Although it was hypothesized why this might have occurred, there appear to be no clear answers. This is concerning, especially if the reason they were underutilized was due to low acceptability. Both exercises use audio for delivery. It is very possible that the exercises were not utilized in the prior study because participants were in locations where they did not want other people to hear these exercises being played aloud. Consideration could be given for making audio exercises usable without sound as well (e.g., words on the screen with an option to play sound if wanted).
- Participants will only receive two mindfulness exercises for the duration of their quit attempt (30 days). There is some concern that boredom/lack of variety could decrease engagement.
- Clear benchmarks are not well-described for each Aim. For instance, Aim 1 #3: How will "high timeliness and craving ratings" be defined? Similarly, although Aims 2 and 3 provide clear outcomes to be assessed, no specific benchmarks are provided (e.g., what % of time will indicate adherence to wearing the smartband; what score will indicate that the interventions are "acceptable").
- There was no rationale provided for why participants over the age of 55 are excluded.
- The compensation that will be provided for the completion of the mindfulness exercises is a concern for two reasons. First, this will confound the calculation of % completed, as money may be a primary motivator for completion; this will limit generalizability to the real-world. Second, the mindful smoking exercise is provided only when a participant smokes, thus those who lapse/relapse will be compensated more money. Compensation for the wearing of equipment/ answering assessments makes sense, as this does not confound treatment receipt/delivery.
- It is recommended to also track how often participants self-initiate features of the app (i.e., view smoking patterns; practice mindfulness exercises), as these behaviors could signal increased engagement and might be related to secondary outcomes.
- Carbon monoxide monitoring will be confirmed via video chat with a researcher. It is not indicated what application will be used for the video chat and its security features (e.g., end-to-end encryption).

5. Environment:

Strengths

- This study will take place remotely, so no on-site resources are needed for in-person visits. All other needed equipment (e.g., smartbands, ability to video conference) and institutional support appear to be easily available to the research team.

Weaknesses

- No major weaknesses noted.

Study Timeline:

Strengths

- Based on previous research by the investigative team, the recruitment and retention goals seem very feasible.

Weaknesses

- Although many of the app features have been developed, they have not been piloted tested in the configuration this study proposes. Time for pilot testing could be incorporated into the timeline, along with extra time to make necessary modifications.
- Given the large amounts of data that will be collected, allocating time for data management prior to analysis is advised.

Protections for Human Subjects:
Acceptable Risks and/or Adequate Protections
- This study poses low risks to participants, and at the same time, has high potential benefit (e.g., access to smoking cessation treatment). Protection against risks are adequately outlined.

Data and Safety Monitoring Plan (Applicable for Clinical Trials Only):
- Acceptable
  - The DSMP is acceptable. It clearly outlines how participants will be monitored, definitions of adverse event risk categories, and grades of risk.

Inclusion of Women, Minorities and Children:
- Sex/Gender: Distribution justified scientifically.
- Race/Ethnicity: Distribution justified scientifically.
- For NIH-Defined Phase III trials, Plans for valid design and analysis: Not applicable.
- Inclusion/Exclusion of Children under 18: Excluding ages <18; justified scientifically.
- See comment in Approach regarding the lack of a rationale for excluding participants over the age of 55. Otherwise, the proposal outlines plans for the inclusions of a diverse population in regards to sex and race/ethnicity.

Vertebrate Animals:
Not Applicable (No Vertebrate Animals)

Biohazards:
Not Applicable (No Biohazards)

Resource Sharing Plans:
Acceptable
- A data sharing plan is included, which outlines how data will be made available to outside investigators.

Budget and Period of Support:
Budget Modifications Recommended (in amount/time)
Recommended budget modifications or possible overlap identified:
- Dr. Brewer's effort is listed as 0%, and he will be available on an as-needed basis. Given the amount of knowledge Dr. Brewer brings to the project, combined with Dr. Garrison's early career status, it is recommended that a specific amount of effort be allocated to Dr. Brewer over the project period. Comments related to expanding the timeline to include pilot testing and data management may require additional time (and additional funding) to be allocated.

CRITIQUE 2

Significance: 1
Investigator(s): 1
Innovation: 2
Approach: 2
Environment: 1

Overall Impact: The goal of this application to create a smartband/smartphone-based mindfulness intervention for smoking cessation. This is a highly innovative project that has the potential to make a strong and sustained impact on the fields of substance use, mindfulness, and public health. The

research team is well-prepared to conduct the study and achieve the aims. The application is very well-written. There are several strengths in the significance, innovation, and approach. Few weaknesses have been identified (e.g., psychometrics and descriptions for measures, Somatix's access to participant data; role/tasks for consultant); however, they do not detract from the potential of this project.

1. Significance:
Strengths
- The Significance section is very well-written and provides a strong rationale for the proposed project.
- The application targets a very serious health problem in the US: smoking.
- The significant impact on the fields of substance use, mindfulness, and public health is likely to be very strong with the addition of a smartband/smartphone app to treat smoking in real-time.
- By clearly assessing the treatment fidelity, adherence, and acceptability of the intervention as part of the proposed study, the results will inform a full-scale clinical trial in the future.
- The findings from the preliminary studies are strong and provide support for the proposed aims.

Weaknesses
- No major weaknesses noted.

2. Investigator(s):
Strengths
- The principal investigator and coo-investigators are well-suited to lead the project and successfully achieve the aims.
- The research team has experience in the areas of smoking cessation, mindfulness, and smartphone apps as well as conducting clinical trials with adults.

Weaknesses
- Dr. Brewer's consultant role and tasks in the project are not listed, nor is his hourly rate for consultation.
- Dr. Brewer is a consultant; however, his role in the project is unclear.

3. Innovation:
Strengths
- The inclusion of a device to detect and track smoking in real time and deliver exercises are part of an intervention is very innovative.
- This application includes the first study examining the feasibility of the proposed three-step intervention. The findings will provide the foundation for a larger scale clinical trial.
- The use of a brief intervention, which is empirically supported, to address a serious health concern.

Weaknesses
- The first three innovations listed in this section are more specific to SmokeBeat and the creator Somatix rather than to the actual project.

4. Approach:
Strengths
- The findings from the proposed study will provide a foundation for a larger study in the future.
- The overview (including the figure) of the intervention, and mindful smoking and RAIN exercises are very clear and well-described.
- The planned recruitment and enrollment seem reasonable and the overall design is feasible.
- The video-based onboarding will likely contribute to the retention of participants.

Weaknesses
- The psychometrics for the measures in Table 1 are not provided. These measures are also not listed in the Outcome Measures section.

- If the User Experience questionnaire and helpfulness ratings are considered primary outcomes in the study, more information about the items and rating scales is needed for both to assess their ability to measure acceptability.
- The research team will exclude someone for making more than one attempt to complete the screening survey. How will the team prepare for bots or fake accounts?
- Will Somatix's staff be trained in Human Subjects or Ethics given that they will have access to participant data from the mobile devices?

5. Environment:
Strengths
- The research team has recruitment strategies in place that have been successful as part of previous studies.
- The facilities at Yale and Somatix will support the proposed trial.

Weaknesses
- No major weaknesses noted.

Study Timeline:
Strengths
- The study timeline for programming the measures and study, recruiting participants, and completing the study seems reasonable.

Weaknesses
- Four months seems like a long time to prepare findings to report on the R01 application.

Protections for Human Subjects:
Acceptable Risks and/or Adequate Protections
- It is unclear whether the personnel at Somatix have any Human Subjects or IRB training given that they will have access to participants' data.
- It is unclear who on the team will be available if a participant has trouble with nicotine withdrawal?

Data and Safety Monitoring Plan (Applicable for Clinical Trials Only):
- Acceptable

Inclusion of Women, Minorities and Children:
- Sex/Gender: Distribution justified scientifically.
- Race/Ethnicity: Distribution justified scientifically.
- For NIH-Defined Phase III trials, Plans for valid design and analysis: Not applicable.
- Inclusion/Exclusion of Children under 18: Excluding ages <18; justified scientifically.

Vertebrate Animals:
Not Applicable (No Vertebrate Animals)

Biohazards:
Not Applicable (No Biohazards)

Resource Sharing Plans:
Acceptable

Budget and Period of Support:
Recommend as Requested

CRITIQUE 3

Significance: 2
Investigator(s): 3
Innovation: 2
Approach: 5
Environment: 1

Overall Impact: The applicants propose a single arm feasibility study in smokers of a smartband to track smoking episodes and to deliver a brief mindfulness intervention to first help them become aware of their smoking patterns and then to assist them in dealing with cravings. The study appears to build off previous work by the applicants and adds multiple novel components to the three-phase intervention. Participants will be recruited nationwide using the internet. Many features of this study design have been tested and refined in previous studies. Despite these excellent design features, there are some concerns about the capacity to recruit and engage a diverse group of smokers in mindfulness, the capacity to recruit individuals with health disparities (who are more likely to smoke), and some concerns about the long term uses of these data.

1. Significance:
Strengths
- Finding novel ways to decrease smoking is exceptionally important, given the relationship between smoking and both mortality and morbidity.
- The use of mindfulness interventions for smoking cessation is somewhat novel and there is evidence that it could be beneficial.
- Apps could be an important way to deliver mindfulness to more people.
- The applicants lay out a logical path to their next trial.

Weaknesses
- This intervention would not be available to people who lack access to smartphones, which could mean it is not accessible to those who have the greatest health disparities.

2. Investigator(s):
Strengths
- The team is experienced with addictions, mindfulness, clinical trials and successful recruitment. In addition, they have experience with prior clinical trials of mindfulness apps for smoking.

Weaknesses
- The principal investigator seems to be doing virtually everything on the study from obtaining Human Subjects assurance through data collection and analysis with very little co-investigator support. A project manager could be appropriate on this project as well as potentially someone with experience related to programming data.

3. Innovation:
Strengths
- The use of wearables to track smoking is novel.
- Automatic detection of smoking would be novel (although people are required to report honestly to validate it).
- Focused capacity to deliver mindfulness “when especially needed”.
- Predicting smoking behavior is novel.

Weaknesses
- Fairly short-term intervention, only 30 days post “quit” is considered a limitation.

4. Approach:
Strengths

- The trial endpoints for feasibility and acceptability are quite reasonable.
- Inclusion and exclusion criteria are reasonable.
- The three-step intervention seems reasonable on its face, though there are questions about the time devoted to steps 2 and 3.
- The capacity to recruit nationwide is positive.

Weaknesses

- It looks like the mindfulness training in their earlier study did not lead to differences in biochemically-verified smoking abstinence at 6 months and overall rates were around 11% (slightly lower in the mindfulness app group). It is unclear why this is so much better that the study is likely to be positive.
- Is there data supporting seven days as a good amount of time for the mindful smoking portion of the intervention? Is 20 days enough time for the “RAIN” portion of the study? If people do choose to smoke after the quit date, should the app help them smoke mindfully during their smoke?
- There was insufficient data on how they will reach out to disadvantaged populations and men provided. These populations have higher rates of smoking. They may also be less interested in using mindfulness techniques.
- No information is provided on the participant burden of the proposed assessments, but it needs to be modest and focused to achieve high response rates.
- It is unclear if the participants will receive any instruction in mindfulness apart from the 2-3-minute exercises from the app. Is this sufficient exposure for participants to learn how to use mindfulness on their own? Or, would they always be dependent on the predictive ability of the app? More information about the exercises is needed.
- Most of the feasibility aims have already been completed in prior studies, so it is not completely clear what features of this study are unique.
- A compelling case has not been made regarding why 200 participants are needed for feasibility.
- It is not clear what the applicants will do next if their data suggest a need for more time to determine smoking profiles, time to quit, etc. as they will only have data from one period (21 days).

5. Environment:
Strengths

- The environment is outstanding.

Weaknesses

- No major weaknesses noted.

Study Timeline:
Strengths

- The timeline is well described and the amount of time for these activities looks reasonable.
- Recruitment looks doable, given successful recruitment for another trial at about twice the monthly rate.

Weaknesses

- Inconsistencies in the recruitment rate and recruitment timeline. The timeline proposes to recruit 25 participants over the course of 9 months – in their timeline vs. 8 months described in the grant (200 to 225 participants), but in the recruitment and retention plan, the applicants claim they will enroll 300 participants to account for estimated high attrition.

Protections for Human Subjects:
Acceptable Risks and/or Adequate Protections

- The Human Subjects section is reasonable.

Data and Safety Monitoring Plan (Applicable for Clinical Trials Only):

- Unacceptable
  - Given the low-risk intervention, there should be an independent monitor, not the study principal investigator, to ensure that the study is proceeding smoothly.

Inclusion of Women, Minorities and Children:
- Sex/Gender: Distribution justified scientifically.
- Race/Ethnicity: Distribution justified scientifically.
- For NIH-Defined Phase III trials, Plans for valid design and analysis: Not applicable.
- Inclusion/Exclusion of Children under 18: Excluding ages <18; justified scientifically.
- These are fine.

Vertebrate Animals:
Not Applicable (No Vertebrate Animals)

Biohazards:
Not Applicable (No Biohazards)

Resource Sharing Plans:
Acceptable

Budget and Period of Support:
Recommend as Requested

CRITIQUE 4

Overall Impact: This application is from a multidisciplinary team who have considerable expertise in clinical trials and mindfulness interventions. The principal investigator is currently on a K12 where she did an RCT with an app. This proposed project builds on that study by including a wearable device to detect smoking patterns. The study addresses a significant problem and is viewed as innovative with the intervention. The aims are appropriate as they will be assessing treatment fidelity, adherence, and acceptability. The study will use a national recruitment via online approaches to enroll 200 people. The design is carefully planned and will involve one group (no randomization) who will receive the intervention. They will wear the armband for 60 days. For most of the aims, specific benchmarks are noted, such as > 80% detection rate. However, a couple outcomes are not specified, but the application states that they expect on average high scores. Potential pitfalls are presented with ways of overcoming them or minimizing their effect. The timeline is feasible and will inform a larger RCT.

Budget and Period of Support:
Budget Modifications Recommended (in amount/time).
Recommended budget modifications or possible overlap identified:
- 75% effort for the principal investigator is extremely high.

THE FOLLOWING SECTIONS WERE PREPARED BY THE SCIENTIFIC REVIEW OFFICER TO SUMMARIZE THE OUTCOME OF DISCUSSIONS OF THE REVIEW COMMITTEE, OR REVIEWERS' WRITTEN CRITIQUES, ON THE FOLLOWING ISSUES:

PROTECTION OF HUMAN SUBJECTS: ACCEPTABLE
The plans for protecting subjects are clearly and appropriately described, however, the panel noted some details missing on the role and training of personnel at Somatix and the availability of team members to address participant's issues with nicotine withdrawal.

INCLUSION OF WOMEN PLAN: ACCEPTABLE

INCLUSION OF MINORITIES PLAN: ACCEPTABLE

INCLUSION OF CHILDREN PLAN: ACCEPTABLE

COMMITTEE BUDGET RECOMMENDATIONS: The justification for the 0% effort of consultant and the 75% effort of the principal investigator was considered inadequate.

Footnotes for 1 R34 AT010365-01; PI Name: GARRISON, Kathleen A.

NIH has modified its policy regarding the receipt of resubmissions (amended applications). See Guide Notice NOT-OD-14-074 at http://grants.nih.gov/grants/guide/notice-files/NOT-OD-14-074.html. The impact/priority score is calculated after discussion of an application by averaging the overall scores (1-9) given by all voting reviewers on the committee and multiplying by 10. The criterion scores are submitted prior to the meeting by the individual reviewers assigned to an application, and are not discussed specifically at the review meeting or calculated into the overall impact score. Some applications also receive a percentile ranking. For details on the review process, see http://grants.nih.gov/grants/peer_review_process.htm#scoring.

MEETING ROSTER

The roster for this review meeting is displayed as an aggregated roster that includes reviewers from multiple AT Special Emphasis Panels Meetings
for the 2019/01 council round.
This roster for AT is available at:
http://public.era.nih.gov/pubroster/Reports?DOCTYPE=SEP&DESFORMAT=PDF&AGENDA_SEQ_NUM_P=354371
